# Hearing Performance in the Follicular-Luteal Phase of the Menstrual Cycle

**DOI:** 10.1155/2018/7276359

**Published:** 2018-08-19

**Authors:** Seyede Faranak Emami, Nasrin Gohari, Hossein Ramezani, Mariam Borzouei

**Affiliations:** ^1^Hearing Disorders Research Center, School of Rehabilitation, Hamadan University of Medical Sciences, Hamadan, Iran; ^2^School of Rehabilitation, Hamadan University of Medical Sciences, Hamadan, Iran; ^3^Department of Audiology, University of Social Welfare and Rehabilitation Sciences, Tehran, Iran

## Abstract

**Introduction:**

Estrogen has a protective role on auditory function. It may have an excitatory action on auditory nerve fibers and can have a neuroprotective effect. Progesterone has a mainly inhibitory action on the central nervous system, which may balance the mainly excitatory action of estrogen.

**Objective:**

To determine changes in hearing performance with pure tone audiometry (PTA), tympanometry, distortion product otoacoustic emissions (DPOAEs), and auditory brainstem responses (ABR) as hormonal changes occur from follicular to luteal phase.

**Materials and Methods:**

Twenty healthy female volunteers (age 19 ± 30 years) with normal menstrual cycle and without any hearing problems are included in this case-control study. Hearing evaluation was performed on the 13th day of the menstrual cycle (follicular phase) and then on the 22nd day (luteal phase).

**Results:**

All of the participants had normal results in follicular phase. In luteal phase, four cases showed abnormalities as follows: reduced hearing thresholds 250 Hz (mean= 15 dBHL), increased amplitudes of DPOAE (mean= 3 dBspl), decreased middle ear pressure (mean= -110 dapa), and delayed ABR interpeak latencies (mean of IPLs I-III= 0.4 and mean of IPLs III-V= 0.6 ms).

**Conclusions:**

In some women, changing of ovarian hormones may induce fluctuating hearing and increased progesterone in luteal phase can lead to abnormal outcomes in auditory function. However, elevated estrogen modifies its consequences in follicular phase.

## 1. Introduction

Estrogen and progesterone are female sex hormones and their secretion change during menstruation. Estrogen (oestrogen) rises during the discharge of the white body or follicular phase [1, 2]. It is responsible for the growth and adjustment of the female reproductive and secondary sex characteristics. There are three major endogenous estrogens in females that have estrogenic hormonal activity: estrone (E1), estradiol (E2), and estriol (E3). Another type of estrogen is called estetrol (E4), which is produced only during pregnancy. Estrogens circulate at lower levels than androgens in both men and women. While estrogen levels are significantly lower in males compared to females, estrogens nevertheless also have important physiological roles in males [1, 3].

Estrogen has a protective role on auditory function. It may have an excitatory action on auditory nerve fibers and can have a neuroprotective effect. At the cochlear level, estrogen receptors alpha and beta have been identified in the inner ear, including spiral ganglion type I cells, the stria vascularis, and cochlear blood vessels. Estrogen may influence auditory transmission, while the receptors in the stria vascularis may affect fluid electrolyte balance in the cochlear fluids and modulating cochlear blood flow [4].

Reduction of estrogen results in poor performance of the auditory pathway. Blocking estrogen receptors alpha and beta had led to a decline in suppression of DPOAEs as a result of a reduction of the medial olivocochlear suppression. This decline could be similar to what occurs with aging and precedes the onset of age related hearing loss, which may suggest that estrogen has a protective role on auditory function, in conjunction with its neuroprotective effect. It has been observed that the decrease in estrogen levels, e.g., after menopause or in Turner's syndrome, is associated with an increased frequency of neurodegenerative disorders. Another proposed mechanism would implicate estrogen as a free radical scavenging antioxidant [5].

Important point is that the entire central nervous system, auditory, and vestibular structures are dimorphism; i.e., there is a gender difference in serotonergic system. It has been reported that there is an increase in 5-HT activity in the female brain compared with the male and a decrease in the whole brain 5-HT synthesis in women compared with men. It was also found to control the gain the inferior colliculus neural responses in a positive or negative direction depending on the type of auditory stimuli [4].

Progesterone is also secreted by the adrenal glands of the central nervous system. It increases in luteal phases of the menstrual cycle, pregnancy, and embryogenesis function. It has a variety of important functions in the body and is also a crucial metabolic intermediate in the production of other endogenous steroids, including the sex hormones and corticosteroids, and plays an important role in brain function as a neurosteroid [2, 5].

Progesterone has a mainly inhibitory action on the central nervous system, which may balance the mainly excitatory action of estrogen. It was found to decrease 5-HT levels and this may affect auditory processing indirectly and may cross-react with other steroid receptors (such as glucocorticoid and mineralocorticoid receptors) present in the cochlea or more proximal areas of the auditory system. It may also influence the auditory system through its interaction with the steroid binding sites on GABA-A receptors acting as a GABA-A agonist, which are present throughout the auditory system [4]. Changes in electrolyte fluid levels caused by high levels of progesterone in the luteal phase can induce a feeling of fullness in the ears, imbalance, tinnitus, or symptoms similar to Meniere's disease [6].

So, this study aimed to determine changes in hearing performance with pure tone audiometry (PTA), tympanometry, distortion product otoacoustic emissions (DPOAEs), and auditory brainstem responses (ABR) as hormonal changes occur from follicular to luteal phase.

## 2. Materials and Methods

The contract for this project was signed on July 22, 2014, with number 9304312272. The sample size consisted of twenty healthy female of age 19–30 years. They studied from July 22, 2014, to July 15, 2015.

Study was case-control; each one was compared with herself and subjected to a two-stage assessment of hearing function by standard clinical audiological battery tests. Assessments started on the 13th day of the menstrual cycle (follicular phase) and then on the 22nd day (luteal phase). The levels of estradiol and progesterone were measured. We chose the people who were sure to be healthy at the first stage of the evaluation. The results of the first assessment phase were recorded as the baseline (control group). In the second stage (luteal phase), changes (possibility of abnormalities) were recorded as case group and compared to the first stage. Ovulation was estimated by measuring basal body temperature (BBT). Subjects were instructed to note down their menstrual calendar for at least 2 months along with their BBT for detecting the day of ovulation. The study was conducted under standard laboratory conditions (room temperature 26 ± 2°C) in sound proof room. This study compared PTA, tympanometry, and DPOAE and ABR between pre- and postovulatory phases of menstrual cycle using Madsen: OB-822, Maico: MI.34, Homoth, and Labat Epic-plus.

Inclusion criteria are as follows: hearing status = normal, gynecological history = normal, body mass index (BMI) = 18.5 to 24.9 kg/m^2^, menstrual cycle = 28 days, level of estradiol for the 13th day in the follicular phase > 600 pg/ml and for the 22nd day in the luteal phase < 250 pg/ml, and level of progesterone for similar days < 50 and > 2000 ng/dl, respectively.

Exclusion criteria are as follows: history of any hearing loss, genital infections, ovarian failure, Turner syndrome, hypothyroidism, Stein-Leutal syndrome, menopause, pregnant or lactating mother, polycystic ovary syndrome, early puberty, receiving steroid hormones, therapy for depression, having history of head injury, stroke, heart attack, disorders of endocrine function, metabolic neoplastic pathologies, anorexia nervosa, liver cellular necrosis, harmful doses of nicotine or alcohol, and alcoholic liver. This information was obtained based on medical records and case history.

Procedure: normal PTA thresholds were in the frequency range of 250–8000 Hz of less than 15 dB [7] (refer to table 3.2 in (7) [7]). Normal tympanometry was middle ear pressure between the limits of ± 50 dapa [8].

In the first stage of the evaluation, it was important for us to select people whose middle ear pressure has a minimum deviation of zero dopa. Normal DPOAE amplitudes were 3 dBspl above the noise floor (an* absent* DPOAE is easy to classify. If the DPOAE is not present at two or more f2 frequencies with sufficient signal-to-noise ratio (SNR), it is an absent response. What is sufficient SNR? This depends on noise floor calculations used by the instrumentation. Some instrumentation calculates the mean noise floor with response variability (standard deviations) incorporated into the measurement. If this is the case, a 3–4 dB SNR is sufficient to detect a response). The f 1/ f 2 ratio was fixed at 1.22 and stimulus levels were held constant at L1; 65 dBspl, and L2; 55 dBspl. The 2f1−f2 DPOAE amplitudes were recorded at frequencies 1.0, 1.5, 2.0, 3.0, 4.0, 6.0, and 8.0 KHz [9, 10]. ABR was tested with electrode arrays: noninverting; high forehead and inverting; ipsilateral and ground; contralateral and stimulus response; 2000, rate; 11.1/s, filters; 70 to 3000 Hz [10].

Data analyses: Kolmogorov-Smirnov and independent test were used. P < 0.05 was considered to indicate statistical significance.

## 3. Findings

Twenty healthy volunteers with normal menstrual cycle and without any hearing problems were included. All of the participants had normal results in follicular phase.

**Figure 1:**
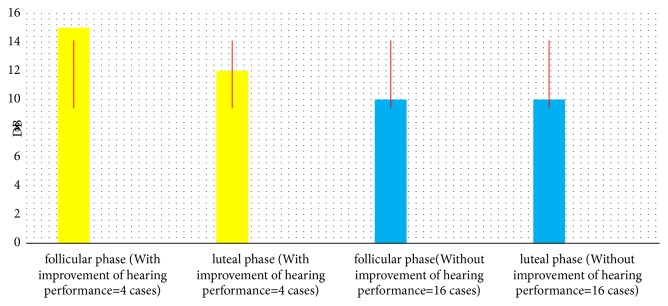
Mean DPOAEs of twenty healthy female in follicular-luteal phase.

**Figure 2:**
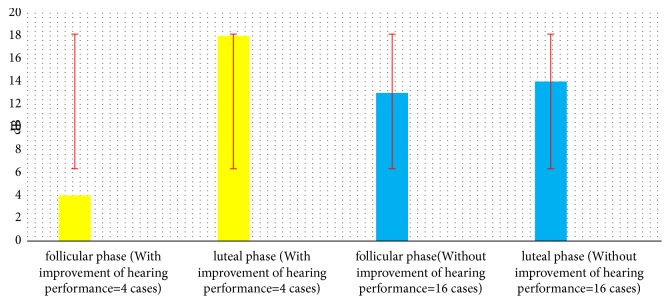
Mean hearing thresholds 250 HZ of twenty healthy female in follicular-luteal phase.

**Figure 3:**
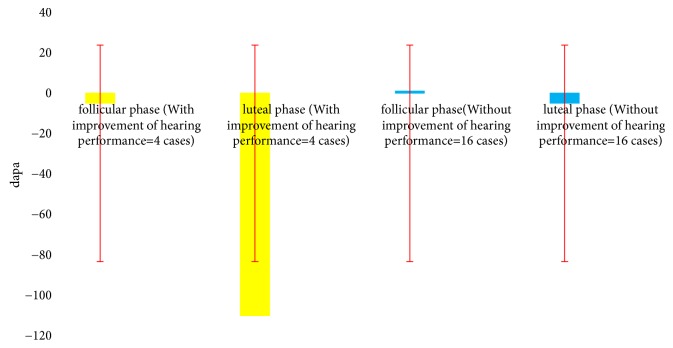
Mean middle ear pressure of twenty healthy female in follicular-luteal phase.

Referring to Tables 1 and 2 and Figures 1, 2, and 3 we can see that, during luteal phase, four cases showed abnormalities as follows: reduced hearing thresholds 250 Hz (mean= 15, min= 10, and max = 20 dBHL), increased amplitudes of DPOAE (mean= 3, min = 1, and max = 5 dBspl), decreased middle ear pressure (mean= 110, min= -90, and max= -150 dapa) (Table 1), and also delayed ABR interpeak latencies (mean of IPLs I-III= 0.4; mean of IPLs III-V= 0.6 ms), (Table 2).

Apart from these four subjects, there are three others (total of seven cases) during the luteal phase complained of a feeling of fullness in the ears, heaviness of the head, and nasal congestion, but they did not have sensation of dizziness or vertigo. All of these seven cases had abnormal results in eustachian tube function test {(difference between the ventilation of the middle ear pressure during Valsalva maneuver in compared to Toynbee maneuver was less than 20 dapa [8]}. All differences were not significant (P > 0.05).

## 4. Discussion

We observed that 20% of our cases had abnormal results in luteal phase (reduced hearing thresholds 250 Hz, increased amplitudes of DPOAE, decreased middle ear pressure, and delayed ABR interpeak latencies). Our results showed that, in some sensitive women, changes in ovarian hormones can lead to abnormal outcomes in auditory tests. The cause of this problem seems to be due to individual differences. It seems that the auditory system in some women is more sensitive to hormone changes. In this group of women, increasing the level of progesterone in luteal phase may partially affect the function of the auditory system.

Our results are consistent with the research of other researchers. It may have a negative effect on both peripheral (DPOAE) and central auditory function and support the positive role of estrogen on hearing function. Also, increase in interstitial fluids (possibly due to progesterone) affects the eustachian tube function and thus may lead to poorer thresholds [4, 11].

Estrogen plays a main role in maintaining the normal hearing of young and middle-aged women in comparison to postmenopausal females [4, 11, 12]. The basal ACTH plasma level seems to rise in the late follicular phase possibly due to the enhancing effect of estrogen on corticotrophin releasing factor gene transcription in the hypothalamus. The rise of ACTH during the late follicular phase is not associated with higher free cortisol level, due to estrogen induced changes in corticosteroid binding protein levels. The lower level of free cortisol may affect the physiological response to stress during this phase of the menstrual cycle. The enhancement of vasopressin secretion is possibly due to estrogen, which was found to increase vasopressin levels in females who had undergone oophorectomy. This may lead to fluid retention or redistribution which occurs in some women in the premenstrual period of the menstrual cycle and may also affect the fluid balance in the cochlea and thus affect auditory function. Estrogen stimulates opioid receptor expression and stabilizes the levels of endorphins that tend to decreases after menopause (surgical or spontaneous). This may be associated with mood changes that can be helped by estrogen treatment, which increases endorphin levels in plasma. Estrogen also affects mood by facilitating the function of enkephalin that is also important for reproductive behavior [4].

Because of the excitatory and neuroprotective role of estrogen, the high tone hearing thresholds in males compared to females are poorer and due to inhibitory role of progesterone, hearing thresholds for low frequencies are weaker in women than their male counterparts [12]. In the middle school age group, auditory thresholds are lower than boys, but differences are not statistically significant. The amount of OAEs is higher and improved in women, the probability of SOAEs (spontaneous) in women is 75%, while in men it is 58% [1, 4]. The amplitude of ABR waves is more pronounced in older adult women with shorter interpeaked times. Similar results are observed in girls' newborns [5].

Also, the shorter ABR latencies during the periovulatory phase of the cycle may suggest that the high estrogen level is associated with shorter ABR latencies. The higher level of estrogen may alter the speed of sensory neurotransmission in the brain stem by modulating glutamate transmission. The higher levels of estrogen may result in an increase in neurosteroids (such as allopregnanolone) which facilitate GABA inhibition in the auditory midbrain. The difference between the ABR latencies in the periovulatory phase and luteal phase would be compatible with progesterone blunting the effect of estrogen, by inhibiting its action in the auditory brainstem [4]. Several studies have been conducted on the effect of estrogen hormone levels on ABR. The higher level of estrogen may alert the speed sensory neurotransmission in brain stem by modulating glutamate transmission. Studies have shown that the delay time of ABR waves becomes more in the postovulation stage [13]. These reports confirm our research results.

Increasing levels of estrogen rises nerve steroids, which facilitates gamma aminobutyric acid (GABA) inhibition in the brain. Estrogen therapy on a species of mice increased the level of allopregnanolone in the central and peripheral level and the difference in latency of the pre- and postmenopausal women with ovarian hormonal changes and the stimulatory and inhibitory effects of estrogen and progesterone can be argued [4]. Changes in the auditory functions have been considered in women who have reached the menopause stage, and they cause hearing losses. Since the onset of hearing loss in women is higher than that of older men, it coincides with the age of menopause [13]. Women, who suffer from premenstrual syndrome, may have changes in their auditory function, which occurs in 5-8% of postmenopausal female with moderate to severe mood, behavior, physics, and occupational or social impairments [2].

Reduction of DPOAE amplitudes following age increases in various species of motility and after menopause. Direct hearing loss due to age is confirmed by pure audiometry and menopause [14]. The low levels of hormones during the premenstrual phase may relate to less sensitive auditory function. Women had fluctuating hearing loss associated with the ovarian cycle that occurred in the late luteal phase and improved after the onset of menses [4]. Based on the report, a lower level of estradiol serum improves hearing sensitivity in postmenopausal women. Estrogen-mediated postmenopausal women have a better hearing sensitivity than women who have not used estrogen [13, 15]. The effectiveness of some drugs has been confirmed in reducing and improving the symptoms of women. For example, docosahexaenoic acid (DHA, 625 mg) can be effective in modulating some perimenopausal symptoms in women and consequently can contribute to improve their quality of life. It seems to have a direct activity on the neuronal conduction time into the audiological system [16]. Another study done in this regard was conducted to determine the effects of the monophasic oral contraceptive containing 30 mcg ethinylestradiol and 3 mg drospirenone on the nasal respiratory epithelium in premenopausal women, which showed that maturation index of the nasal respiratory epithelium seems to depend on the variation of the ovarian steroids during the menstrual cycle and on the iatrogenic effects of oral contraception [17].

Studies show that women with premenstrual syndrome (PMS) have had irregular responses in the presence of stress compared to noninfected women. These changes in PMS women have a potential impact on auditory function [18]. Changes in auditory function were detected based on the ABR test, which indicated that women with PMS had a longer delayed time than noninfected women [3]. Women with severe PMS have a longer latency of III and V waves, while women with PMS have a wavelength of III wavelengths compared to noninfected women. The difference in ABR times can be due to neurological abnormalities in the brain stem [11, 19]. Additionally, in women with PMS, wave delay time III was longer than the control group. The presence of these differences in ABR between women with and without PMS indicates a neurobiological tendency to PMS [4, 19].

Contraceptive drugs inhibit the levels of estrogenic hormone levels. In women, who have contraceptive medications compared to women who have not, the range of OAEs is smaller and the responses are weaker and similar to men [13, 20].

The physiological changes in pregnancy may affect auditory function mimicking the auditory dysfunction seen in Meniere's disease, i.e., low frequency elevated thresholds, with lowering of uncomfortable loudness levels. The higher levels of ovarian hormones in pregnancy may shorten ABR wave latencies, but the higher level of progesterone may blunt this effect. Neural conduction in the brain stem may be slower during pregnancy due to the fact that higher levels of steroids that facilitate GABA inhibition the absolute wave latencies were slightly shorter but did not reach significance in eight pregnant women in the third trimester compared with age-matched nonpregnant women. However, the I–III, III–V, and I–V interpeak latencies which indicate neural conduction were significantly longer in pregnant women [4].

## 5. Conclusion

The auditory system seems to be sensitive to changing female hormones in some women and that is important factor, which can be the reason for reinforcement-attenuation of responses. It can affect the latencies of auditory brainstem responses, the amplitudes of distortion product otoacoustic emissions, the pressure of middle ear, the function of eustachian tube, and low frequency hearing thresholds.

## Figures and Tables

**Table 1:** Mean and standard deviation of physiological audiology tests of twenty healthy female in follicular-luteal phase.

		*follicular phase*	*Luteal phase*	*follicular phase*	*Luteal phase*	*follicular phase*	*Luteal phase*
Subject	N	DPOAEs (dBspl)	DPOAEs (dBspl)	Hearing thresholds (dBHL) 250 HZ	Hearing thresholds (dBHL) 250 HZ	middle-ear pressure (dapa)	middle-ear pressure (dapa)
With improvement of hearing performance	4	*15 *± 0.65	*12 *± 0.30	*3.75*± 0.21	*17.78* ± 0.89	*-5* ± 0.17	-110 ± 0.25

Without improvement of hearing performance	16	10 ± 0.85	10 ± 0.60	12.45 ± 0.45	13.32 ± 0.21	1 ± 0.10	-5 ± 0.15

**Table 2:** Mean and standard deviation of auditory brainstem response (ABR) interpeak latencies (IPLs) of twenty healthy female in follicular-luteal phase.

		*follicular phase*	*luteal phase*	*follicular phase*	*luteal phase*
Subject	n	IPL of I-III(ms)	IPL of I-III(ms)	IPL of III-V(ms)	IPL of III-V(ms)
With improvement of hearing performance	4	2.51± 2.65	2.55± 2.30	2.62± 0.61	2.68 ± 0.89

Without improvement of hearing performance	16	2.51± 1.90	2.50± 2.13	2.63 ± 0.45	2.64 ± 0.21

## Data Availability

The data used to support the findings of this study are available from the corresponding author upon request.

## Additional Points

*Methodological Consideration/Limitations. *The very important thing was to perform an activity at a specific time and in line with the menstrual cycle of the women, which created limitations in the loss of research patterns or unwillingness to continue the assessment.* Ethical Considerations*. In this research, privacy and personal information were reserved and respected. There was no action that was in conflict with their safety, health, and well-being and they were excluded from the survey at any stage that the individuals concerned were reluctant to continue to cooperate.

## References

[1] Stanczyk F. Z. (2002). Pharmacokinetics and potency of progestins used for hormone replacement therapy and contraception. *Reviews in Endocrine and Metabolic Disorders*.

[2] Burger H. G. (2002). Androgen production in women. *Fertility and Sterility*.

[3] Charitidi K., Meltser I., Tahera Y., Canlon B. (2009). Functional responses of estrogen receptors in the male and female auditory system. *Hearing Research*.

[4] Al-Mana D., Ceranic B., Djahanbakhch O., Luxon L. M. (2008). Hormones and the auditory system: A review of physiology and pathophysiology. *Neuroscience*.

[5] Bhatt I., Phillips S., Richter S. (2016). A polymorphism in human estrogen-related receptor beta (ESRR*β*) predicts audiometric temporary threshold shift. *International Journal of Audiology*.

[6] Caruso S., Cianci A., Grasso D. (2000). Auditory brainstem response in postmenopausal women treated with hormone replacement therapy: A pilot study. *Menopause*.

[7] Robert S. S., Nelson P., Katz J., chashin M., English K., Hood L. J., Tillery K. L. (2015). Pure tone evaluation. *Hand Book of Clinical Audiology*.

[8] Fowllff C. G., Shanks E. G., Katz J., Medwetsky L., Burkard R. (2002). Tmpanometry. *Hand Book of Clinical Audiology*.

[9] Abdala C., Visser-Dumont L. (2001). Distortion product otoacoustic emissions: A tool for hearing assessment and scientific study. *Volta Review*.

[10] Hood L. J., Burkard R. F., Eggermont J. J., Don M. (2007). Auditory Neuropathy and Dys-synchrony. *Auditory Evoked Potentials Basic Principles and Clinical Application*.

[11] Da Silva Souza D., Luckwu B., De Andrade W. T. L., De Figueiredo Pessoa L. S., Do Nascimento J. A., Da Rosa M. R. D. (2017). Variation in the hearing threshold in women during the menstrual cycle. *International Archives of Otorhinolaryngology*.

[12] Ors C. H., Karaman H. I. O. (2018). Investigation of auditory potentials and cognitive impairment in premenstrual syndrome. *Neurological Sciences*.

[13] Curhan S. G., Eliassen A. H., Eavey R. D., Wang M., Lin B. M., Curhan G. C. (2017). Menopause and postmenopausal hormone therapy and risk of hearing loss. *Menopause*.

[14] Frisina R. D., Frisina D. R. (2013). Physiological and neurobiological bases of age-related hearing loss: Biotherapeutic implications. *American Journal of Audiology*.

[15] Prabhu P., Banerjee N., Anil A., Abdulla A. (2016). Role of sex hormones produced during menstrual cycle on brainstem encoding of speech stimulus. *European Archives of Oto-Rhino-Laryngology*.

[16] Cianci A., Maiolino L., Giunta G., Rapisarda A. M. C., Di Mauro P., Caruso S. (2017). Neurovegetative disorders of perimenopausal women treated with docosahexaenoic acid (DHA, 625 mg). *Gynecological Endocrinology*.

[17] Caruso S., Serra A., Grillo C., Agnello C., Di Mari L., Cianci A. (2008). Prospective study on the cytological aspects of the nasal respiratory epithelium in premenopausal women taking 30 mcg ethinylestradiol and 3 mg drospirenone oral contraceptive. *Contraception*.

[18] Schilit S. L. P., Currall B. B., Yao R. (2016). Estrogen-related receptor gamma implicated in a phenotype including hearing loss and mild developmental delay. *European Journal of Human Genetics*.

[19] Karlsson S., Henningsson S., Hovey D. (2016). Social memory associated with estrogen receptor polymorphisms in women. *Social Cognitive and Affective Neuroscience*.

[20] Burkard R., Secor S., Katz J. (2002). Overview of auditory evoked potentials. *Hand Book of Clinical Audiology*.

